# Conscientious objection in pharmacist codes of ethics: An international comparison through document analysis

**DOI:** 10.1016/j.rcsop.2025.100609

**Published:** 2025-04-30

**Authors:** L.S. Wong, S.L. Scahill, E. Barton, X.Y. Lim, J. Hikaka, J. Boey, D.J. Exeter, M. Hudson, A. Nu'u, Sanyogita (Sanya) Ram

**Affiliations:** M&HS Building 505 - Bldg 505, 85 Park Rd, Grafton, Auckland 1023, New Zealand

**Keywords:** Document analysis, Code of ethics, Conscientious objection, Pharmacy, International comparison

## Abstract

**Background:**

Conscientious objection (CO) in pharmacy refers to the refusal to provide certain services based on moral or religious beliefs. Person-centred care helps to carve a way forward in balancing the duality of private conscience and public role expectations of the pharmacist. While individual conscience is a factor, pharmacists must also adhere to professional, legal, and regulatory standards. This interplay highlights the need for clear, context-sensitive guidance for both pharmacists and patients to ensure equitable access to services.

**Objective:**

This review aimed to explore and understand the similarities, differences, and limits across international pharmacist codes of ethics in relation to CO clauses.

**Methods:**

The document search focused on a list of OECD member countries. The International Federation of Pharmacists (FIP) website assisted with the identification of relevant regulatory pharmacist organisations (POs) within OECD countries. Information on Codes of Ethics and CO clauses published in English were gathered from POs' websites using specific keywords. Document analysis was employed to qualitatively examine individual Codes of Ethics.

**Results:**

A survey of OECD countries (*n* = 38) identified 96 relevant documents pertaining to pharmacist Codes of Ethics or legislation on CO. Of these, 24 Codes of Ethics in English were identified, 12 of which explicitly mentioned CO. Among these, nine explicitly permitted CO, while six inferred it through moral, religious, or personal grounds. Most (*n* = 11) emphasized the importance of maintaining continuity of care to ensure patient access to services.

**Conclusion:**

There are similarities and differences in Codes of Ethics governing pharmacists' CO worldwide, suggesting variability in practice norms. Consistent guidance across jurisdictions is needed to safeguard patients' rights to access treatment. Future studies on how pharmacists apply ethical codes in CO scenarios could provide valuable insights for updating professional regulatory standards.

## 1 Introduction

Conscientious objection (CO) is the refusal to participate in an activity that an individual considers incompatible with their religious or moral beliefs. The insight, judgement, and actions of pharmacists in the workplace are governed by their professional obligations. Guides, standards and codes help outline conduct and behaviour and quality in practice.^1^ Codes of conduct, and Codes of Ethics for the profession serve as public declarations of the principles and ethical standards that govern pharmacists. It underpins the practice of pharmacists' professional practice, their principles and values. Pharmacists, like other professionals, are not devoid of personal conscience. Pharmacists' philosophy of the world may also be shaped by their upbringing, life experiences, cultural or religious norms, and values of loved ones or those they spend the most time around.^2^ CO clauses are often included in Codes of Ethics to provide a framework for addressing professional conduct in relation to CO.

There is significant professional tension around CO and in articulating the professional and ethical values to which all pharmacists conform. In the United States of America, since the Dobbs v. Jackson Women's Health Organisation decision held that there is no federal constitutional right to abortion, the reversal of Roe v. Wade and Planned Parenthood v. Casey have significantly altered the landscape of abortion provisions in the US.^3^^,^^4^ The removal of the constitutional protection of the right, shifts the focus to professional standards governing what is permissible in healthcare practice and the role of professionals in overseeing the exercise of CO in the provision of services.^2^ It highlights the responsibility of professional organisations to ensure that professional obligations in relation to the provision of services are clearly articulated to the profession.

In a survey, conducted in the USA, 7.5 % of pharmacists surveyed were unwilling to dispense emergency contraception.^5^ An even higher proportion (17.2 %) were unwilling to dispense medical abortifacients.^5^ CO clauses aim to provide a balanced solution to what is considered an inherently complex cultural, social and health related ethical issue. This area of healthcare has provided a rich platform for intense debate on the acceptability and limits of CO within pharmacy and the healthcare environment. At the heart of this healthcare debate is the interconnect and conflict between patient autonomy and equity of access to health services, the personal conscience of pharmacists, and professional guidance. Arguments in favour of the right to object, include a pharmacist's right to exercise independent judgement since they are undertaking service provision personally for the patient.^2^ CO enables both healthcare professionals and patients to mutually respect and tolerate each other's diversity within the context of health service provision. Person-centred care helps to carve a way forward in balancing the duality of private conscience and public role expectations of the pharmacist, where patients' and healthcare providers show tolerance of each other's diverse viewpoints.^6^ The difficulty that arises is in establishing the limits of what is reasonable and acceptable within CO clauses in codes of ethics.

Behavioural norms within a profession are delineated through established guidance that comes in the form of founding documents, which include a Code of Ethics and Standards of Practice or Competency Standards. These documents articulate the expectations for professional conduct and can typically extend beyond the minimum legal and societal standards.^7^^,^^8^ A Code of Ethics outlines the core principles that guide the practice of pharmacy, while Standards of Practice or Competency Standards define the specific performance expectations and professional duties. These documents collectively provide the framework for a comprehensive structure for ethical and moral conduct essential to the responsible execution of professional responsibilities^7^^,^^8^.

Cultural context may influence pharmacists' interpretation of CO. Understanding the role of religion and its influence on societal norms is crucial in explaining the divergent approaches to CO across different jurisdictions.^9^^,^^10^There is a need for clear limits and guidance to ensure that patients receive accessible and unbiased care.^2^ It is accepted that CO clauses are not unfettered and are subject to reasonable limits imposed by the profession and society. This ambiguity has the potential to create confusion and variability over jurisdictions and between professions which ultimately could affect the care a patient receives, affecting social and health equity.^11^ For vulnerable patients such as those seeking gender affirming therapy or end of life care, CO can disproportionately affect access to health services and the restriction of access to care can consequently undermine patient autonomy, in addition to the principles of beneficence and justice.^12^

The literature concerning the existence of CO clauses in pharmacy practice is notably sparse, particularly in terms of the guidance provided to pharmacists. Additionally, there remains a significant gap in understanding the international context of CO clauses as articulated within different pharmacist codes of ethics of different jurisdictions. This lack of comprehensive analysis highlights the need for further investigation into both national and international frameworks governing ethical practices in pharmacy.

### 1.1 Aims

This research aimed to explore international pharmacist codes of ethics in relation to CO clauses. The aim is to describe the similarities, differences and limits that lie across a range of countries or jurisdictions.

## 2 Methods

The foundation for the document search was a list of nations derived from member countries of the Organisation for Economic Co-operation and Development (OECD). The International Federation of Pharmacists (FIP) website's list of member organisations was used to identify and confirm professional and regulatory pharmacist organisations (POs) within the OECD nations. Depending on the information freely provided on their websites, organisations were classified as either professional or regulatory. Professional organisations were those that represented pharmacists and advocated for advancements for their pharmacy workforce, whereas regulatory organisations were invested in registration of the workforce, pharmacist certification or the maintenance of competence of pharmacists, dealt with pharmacist complaints and were involved with the production and distribution of guidance material for the profession in addition to monitoring continuing professional development. The inclusion of FIP as a source of information was due to its international recognition and leadership as a representative organisation for pharmacists. FIP has a partnership of collaborations with United Nations Educational, Scientific and Cultural Organisation (UNESCO) and the World Health Organisation (WHO).

Information about professional code of ethics, and CO clauses, and associated meta data about POs was obtained directly from the POs website. Only freely accessible, public information was included. Keywords searched were “pharmacy” in combination with “code of ethics”, “code of conduct”, “ethics”, “guidelines” and “standards”. “Conscientious Objection” was excluded from the search strategy as this study focused specifically on identifying relevant information within codes of ethics, rather than on separately issued guidance that is not included within foundational documents for pharmacy practice.

Meta data included information about the Code of Ethics relating to pharmacist practice. All meta data, except for FIP membership, was retrieved directly from the PO website. Meta data extracted included country and, if relevant, the state, regulatory authority, document name, year of publication and if there was the presence or absence of a CO or morality clause. Screening was undertaken to remove meta data that did not meet inclusion criteria. Meta data screened for CoE exclusion included jurisdictions where there was no Code of Ethics or a non-English website. If a jurisdiction did not meet the inclusion criteria, its records were excluded and a note to indicate this was added. Some documents did not reference or define CO but inferred it through guidance around abortion or a morality clause; as this was not reflected in a code of ethics, screening removed these documents. Jurisdictions that did not have a unifying code that governed all pharmacists across community, hospital and industry were also removed. Document analysis was used to qualitatively screen the Codes of Ethics based on a pre-developed data collection form.^13^ The search, screening, data extraction and identification were undertaken by two investigators and discussed with a senior investigator. Thematic sorting of the documents was undertaken by one investigator and finalised with a senior investigator.

## 3 Results

**Fig. 1:**
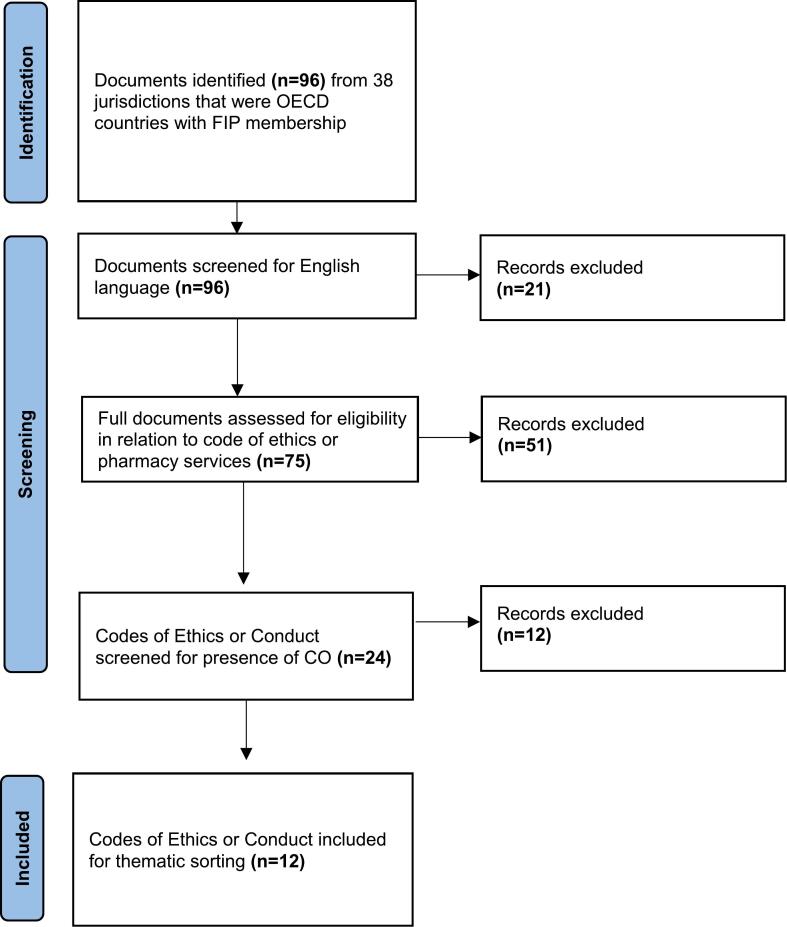
Flow diagram of document inclusion.

Organisations from 38 OECD countries were surveyed, with 96 documents identified for preliminary screening. A final 12 documents were considered to have met the inclusion criteria for further analysis and thematic sorting on CO clauses. Fig. 1 illustrates the document inclusion and exclusion process. Table 1 describes the jurisdictions with Codes of Ethics available that were screened for the absence or presence of CO or addresses CO (*n* = 24). Table 2 shows the delineation of CO clauses or similar within documents that met the inclusion criteria for thematic analysis (*n* = 12).

**Table 1:** Jurisdictions from the OECD that include or imply CO in their document.

Country	State	Regulatory Authority	Document Name	Publication Year	CO present
Australia	N/A	AHPRA: Australian Health Practitioner Regulation Agency	Code of Ethics for Pharmacists (Pharmaceutical Society of Australia)	2017	Yes
Canada	Ontario	Ontario College of Pharmacists	The Ontario College of Pharmacists Code of Ethics	2015	Yes
	Alberta	Alberta College of Pharmacy	Alberta College of Pharmacy Code of Ethics	2009	Yes
	Nova Scotia	Nova Scotia College of Pharmacists (NSCP)	The Code of Ethics of the Nova Scotia College of Pharmacists	2016	Yes
	Manitoba	The College of Pharmacists of Manitoba (CPhM)	Code of Ethics	2012	Yes
	British Columbia	College of Pharmacists of British Columbia	Code of Ethics -College of Pharmacists of British Columbia	2016	Yes
	New Foundland & Labrador	Newfoundland and Labrador Pharmacy Board	Code of Ethics	2014	Yes
	Prince Edward Island	Prince Edward island College of Pharmacists	Code of Ethics	2017	Yes
	Saskatchewan	Saskatchen college of pharmacy professionals	Code of Ethics	2017	No
	New Brunswick	The New Brunswick College of Pharmacists	Code of Ethics	2020	Yes
Estonia	N/A	National Register of Health Care Professionals	Code of Professional Ethics of Estonian Pharmacists	2000	No
Ireland	N/A	The Pharmacy Regulator (Pharmaceutical Society of Ireland)	PSI Code of Conduct	2019	Yes
Netherlands	N/A	Koninklijke Nederlandse Maatschappij ter bevordering der Pharmacie, KNMP	Charter Professionalism of the Pharmacist: Foundation for acting professionally and ethically	2018	No
New Zealand	N/A	Pharmacy Council of New Zealand	Code of Ethics	2018	Yes
Slovak Republic	N/A	Unknown	Act No. 576/2004 on health care and on services related to health care.	2004	Yes
Slovenia	N/A	Slovenian Pharmaceutical Society	Health Services Act	1992	Yes
United Kingdom (England, Scotland, Wales)	N/A	The General Pharmaceutical Council (GPhC)	The Code of Ethics for Great Britain	2001	Yes
United States of America	California	California State Board of Pharmacy	Code of Ethics	2021	No
	Maryland	Maryland Board of Pharmacy	Code of Ethics for Pharmacists	1994	No
	Massachusetts	Massachusetts board of registration in pharmacy	247 CMR 9.00: Code for professional conduct; professional standards for registered pharmacists, pharmacies, and pharmacy departments	2014	No
	New Hampshire	NH office of professional licensure and certification	FP 2024–76, Ph 501 Code of Ethics for Pharmacist	2024	No
	North Carolina	North Carolina Board-Pharmacy	Code of Professional Ethics and North Carolina Board of Pharmacy policies	2011	No
	Ohio	State of Ohio Board of Pharmacy	Code of Ethics for Pharmacists	2021	No
	Washington	Washington state department of health	Code of Ethics for Pharmacists	2021	No

**Table 2:** CO in pharmacist code of ethics from jurisdictions which allow this.

Regulatory Authority	Document Name	Publication Year	CO clauses from Code of Ethics
Ahpra: Australian Health Practitioner Regulation Agency	Code of Ethics for Pharmacists (Pharmaceutical Society of Australia)	2017	“informs the patient when exercising the right to decline provision of certain forms of health care based on the individual pharmacist's conscientious objection*, and in such circumstances, appropriately facilitates continuity of care for the patient.”
Ontario College of Pharmacists	The Ontario College of Pharmacists Code of Ethics	2015	“Members must, in circumstances where they are unwilling to provide a product or service to a patient on the basis of moral or religious grounds, ensure the following: i. that the member does not directly convey their conscientious objection to the patient; ii. that the member participates in a system designed to respect the patient's right to receive products and services requested; iii. That there is an alternative provider available to enable the patient to obtain the requested product or service, which minimizes inconvenience or suffering to the patient.”
Alberta College of Pharmacy	Alberta College of Pharmacy Code of Ethics	2009	“Assist each patient to obtain appropriate pharmacy services from another pharmacist or health professional within a timeframe fitting the patient's needs if I am unable to provide the pharmacy service or will not provide the service due to a conscientious objection. Arrange the condition of my practice so that the care of my patients will not be jeopardized when I will not provide certain pharmacy services due to a conscientious objection”
Nova Scotia College of Pharmacists (NSCP)	The Code of Ethics of the Nova Scotia College of Pharmacists	2016	“Registrants, who do not provide medicines or services to patients because of a conscientious objection including personal, moral or religious reasons, inform pharmacy management of their objections at the earliest opportunity. Pharmacy management provides reasonable accommodation of the registrant's right of conscience and develops an appropriate means to ensure the medicines or services are provided in as timely and convenient a manner as possible”
The College of Pharmacists of Manitoba (CPhM)	Code of Ethics	2012	“Arrange practice to ensure that patients are able to obtain services from another pharmacist or pharmacy in a reasonable time frame if unable to provide the pharmacy service or unwilling to provide the service due to conscientious objection.”
College of Pharmacists of British Columbia	Code of Ethics -College of Pharmacists of British Columbia	2016	“Registrants must provide pharmacy services requested by patients and may only refuse to provide these services for any of the following reasons… the provision of the product or service is contrary to the sincerely held conscientious or religious belief of a registrant, in which case the registrant must ensure that: they have informed and explained to the pharmacy manager and employer of their conscientious or religious belief before they accept employment; if the belief is formed after employment is accepted, they inform the pharmacy manager and employer at the earliest opportunity; they do not discuss their personal beliefs or ask patients to disclose or justify their own beliefs; they participate in a process designed to exercise their freedom of conscience and religion in a manner that respects the patient's right to receive products and services in a timely manner and in a way that minimizes suffering and hardship to the patient”
Newfoundland and Labrador Pharmacy Board	Code of Ethics	2014	“Registrants who are unable to provide medications or services to patients due to a conscientious objection, including personal, moral, or religious reasons, inform pharmacy management of their objections at the earliest possible opportunity, and ensure that each patient receives assistance in obtaining the medication or service from another pharmacist or health professional within a timeframe fitting the patient's needs”
Prince Edward island College of Pharmacists	Code of Ethics	2017	“Make arrangements in practice so that the care of patients will not be jeopardized when exercising conscientious objection.”
The New Brunswick College of Pharmacists	Code of Ethics	2020	“A conscientious objection is an opinion held by a professional that precludes participation in the delivery of an aspect of patient care. Pharmacy professionals may hold sincere beliefs of a conscientious nature that prevent the registrant from performing certain actions. If the service is permitted legally and prescribed by an authorized prescriber, then a claim to conscientious objection means that, ‘but for’, the conscientious objection the registrant would normally have a duty to provide the service.”
The Pharmacy Regulator (Pharmaceutical Society of Ireland)	PSI Code of Conduct	2019	“Must refer patients to an alternative provider if you cannot provide a professional service or medicinal product, including in the case of conscientious objection, ensuring that patient care is not jeopardized or compromised”
Pharmacy Council of New Zealand	Code of Ethics	2018	“Refers patients to alternative providers if personal moral or religious beliefs prevent the pharmacist from providing a professional service, and appropriately facilitates continuity of care.”
The General Pharmaceutical Council (GPhC)	The Code of Ethics for Great Britain	2001	“before accepting employment pharmacists must disclose any factors which may affect their ability to provide services. Where pharmacists' religious beliefs or personal convictions prevent them from providing a service they must not condemn or criticise the patient and they or a member of staff must advise the patient of alternative sources for the service requested”

**Fig. 2:**
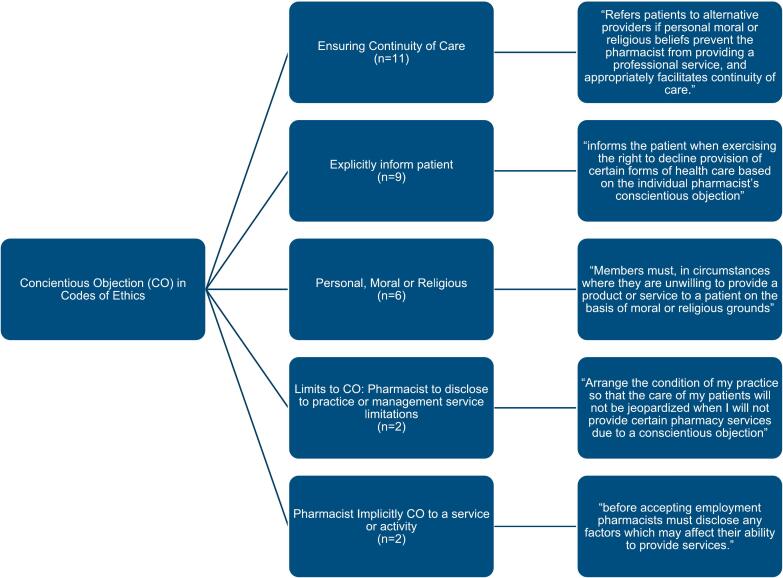
Thematic analysis of CO clauses or inferences with example quotes from code of ethics that are conveyed from the regulator in jurisdictions that allow for this.

The thematic analysis in Fig. 2 demonstrates heterogeneity between the documents in the way that CO is conveyed for pharmacists working within these jurisdictions. Most of the documents indicated that despite explicitly recognising a professional's action may be influenced by CO (*n* = 9) or inferring objection to service provision by moral, religious or personal means (*n* = 6), continuity of care provision remains important, so patients can continue to access health related services (*n* = 11).

Of the jurisdictions that have reference to CO, implied or explicit in their Code of Ethics, only the Australian and New Brunswick Code of Ethics included a specific definition of conscientious objection. Australia defines this as “In health care, involves a practitioner's refusal to engage or provide a service primarily because the action would violate their deeply held moral or ethical value about right and wrong”, whereas New Brunswick states “A conscientious objection is an opinion held by a professional that precludes participation in the delivery of an aspect of patient care. Pharmacy professionals may hold sincere beliefs of a conscientious nature that prevent the registrant from performing certain actions.” Australia focuses on refusal to engage or provide because of moral or ethical values, whereas New Brunswick focuses less on moral or ethical views and more on opinions of an individual.

Codes of Ethics that permitted CO required pharmacists to explicitly inform patients when exercising their right to decline the provision of certain healthcare services based on their individual conscientious objection, or similar language. In contrast, Codes of Ethics that implicitly allowed CO emphasized the need for pharmacists to disclose their CO prior to accepting employment or to a pharmacy manager, rather than directly to patients. In both explicit and implicit cases, the responsibility for disclosing CO resided with the pharmacist, either to the employer or the patient, and did not necessarily require the pharmacist to refer the patient to another appropriate provider. Fig. 2 further illustrates that, thematically, all the Codes of Ethics combine explicit, implicit, and conditional approaches to the exercise of CO rights, provided there is a referral or transfer of care to another provider or pharmacist who does not hold personal, moral, or religious objections to providing the service.

All states in Australia and most provinces in Canada explicitly recognise that a pharmacist's actions may be influenced by CO, whereas in New Zealand and Great Britain, the approach is more implicit. In these latter countries, the Codes suggest that pharmacists may object on personal, moral, or religious grounds, rather than directly stating that services may be withheld due to the exercise of CO. In contrast, while both Canada and Australia recognise that CO may be exercised implicitly, they also provide provisions for explicit objection without requiring justification, provided that the objection is disclosed to a manager prior to employment or accompanied by a referral to another provider who does not share that objection.

There was an overall paucity of Code of Ethics that identified or defined CO specifically across the 38 OECD jurisdictions which were surveyed. In jurisdictions where their CO was included, relevant information was provided through a Code of Ethics issued by the regulatory bodies of pharmacists. In larger countries, pharmacy regulations are administered by distinct jurisdictions, each with its own Code of Ethics. Consequently, a unified ethical framework does not exist across these jurisdictions, given the variability in regulatory structures in operation.

## 4 Discussion

This research examines a diverse collection of Codes of Ethics pertaining to CO within the pharmacy profession. These codes underpin the actions expected to be taken by pharmacists when exercising their right to abstain from providing health services. Codes of Ethics ensure pharmacists adhere to established behavioural norms, provide a framework for evaluating professional conduct in complaints and disciplinary cases, and contribute to professional performance and patient outcomes by standardizing practice and shaping patient experiences.

When situations of CO arise, they often present complex ethical challenges that require pharmacists to carefully reconcile their personal and professional responsibilities. The application of bioethical principles, such as non-maleficence, justice, and beneficence, are often invoked to help pharmacists navigate the tensions and dilemmas. It is most effective when these principles are clearly articulated, consistently applied, and detailed in a Code of Ethics in sufficient depth to provide guidance.^12^^,^^14^

The contemporary and growing roles of pharmacists in general practice settings, in addition to the proliferation of enhanced pharmacy services and the availability of these services are of concern where there is no specific or consistent guidance from regulatory authorities or professional societies about how to manage CO within the profession.14., 15., 16 For instance, New Brunswick offers comprehensive guidance on a conscientious objection action plan, which is enacted when a patient requires care that conflicts with a pharmacist's sincerely held and conscientious beliefs.^17^ In contrast, other jurisdictions lack similar provisions.

Within larger jurisdictions such as Canada or the USA, different states are regulated by an individual professional regulatory authority with its own codes for a variety of reasons including differing state legislation on social issues. These findings indicate that there are notable similarities in addition to differences among the CO guidance that pharmacists worldwide are provided, which suggests behaviour norms concerning CO vary. This variability is consistent with the understanding that the regulation of the pharmacy profession differs across various jurisdictions worldwide. A comparative paper which surveyed professional Codes of Ethics for pharmacists across Portugal, Lithuania and Turkey noted Codes of Ethics were influenced by regional culture and common ethical principles across Europe, however it was noted that there was a need to harmonize these standards across Europe to improve the quality of pharmacy practice across borders.^18^, ^19^, ^20^

Within the USA, researchers found that since 2006, eleven states had CO clauses present in their pharmacy administrative code.^10^ They noted this was double the number from previous research.^10^ They also report that imprecise language throughout the nation allowed rights for patients to vary widely, which in turn meant that access to services also varied depending on the reason and method by which practitioners opted to object to service provision. ^10^ The European Court of Human Rights advises that pharmacists should prioritize their professional responsibilities over their personal beliefs.^21^ Similarly, the American Pharmacists Association's Code of Ethics highlights the significance of respecting each patient's autonomy and dignity.^22^ They emphasize the need to honour personal and cultural differences, grounded in moral obligations and virtues.

The International Pharmaceutical Federation (FIP) state that professional standards in relation to Code of Ethics for pharmacists should include obligations around pharmacists being able “to respect patients' rights and recognise and respect the cultural differences, beliefs and values of patients, carers and other healthcare professionals, particularly in the event of conflict with their own moral or religious beliefs; and to ensure continuity of care for the patient in the event of conflict with their own moral or religious beliefs, based on respect for patient autonomy”.^23^ As noted alongside the previous Codes of Ethics mentioned, there is no specific requirement to include a definition of CO; rather, it is implicitly suggested, with elements loosely implied in the description. For some regions where there is no CO clause or similar, the implications of this could potentially compromise patient care and undermine the trust in the profession from a public health perspective.

Despite the provision of bioethical modelling to assist with decision making and the guidance of a Code of Ethics, pharmacists may still be driven by their personal beliefs.^10^^,^^12^^,^^14^^,^^24^^,^^25^ Evidence-based regulation could play a critical role in mitigating the risks of CO in patient care; however, the extent to which such regulation alone can effectively address its impact on patient outcomes and access, merits further investigation. Exploring how pharmacists apply their Code of Ethics in the exercise of CO within the practice environment could offer valuable insights for professional regulators when revising ethical guidelines for the profession.^26^

New Zealand, Canada, Great Britian and Australia have similar regulatory approaches for regulating pharmacists, however their approach to CO differs. The New Zealand pharmacist Code of Ethics 2018 has no obvious definition of CO. The code leans heavily on morality or religion as the basis for referral to ensure continuity of care. This lack of definition or ambiguity could cause confusion and differences in interpretation and application. Some states in Canada and Great Britain follow similarly and have less explicit clauses, however as there is no specific definition, this may lead to ambiguity on what services practitioners are referring to when they choose to opt out of service provision.

The Australian Code of Ethics, explicitly defines CO as “a practitioner's refusal to provide a service primarily because the action would violate their moral or ethical values”.^27^ Australia's explicit definition of CO provides a more structured framework for pharmacists, leading to clearer expectations and potentially fewer ethical and legal complications compared to the more flexible, less-defined approach in New Zealand.

Great Britain and Ontario implicitly highlight that CO is a pharmacist's responsibility to disclose and that this disclosure happens in the pre-employment phase and they have a referral procedure in place. Where CO is less explicit, pharmacists can more openly interpret how they disclose or practice with their CO in mind. While this study did not quantify adherence to Codes of Ethics, the lack of explicit guidance regarding CO may result in pharmacists increasingly relying on their personal beliefs to inform decision-making on these issues.^28^ This growing reliance on personal beliefs poses the risk that, over time, pharmacists may prioritize these personal convictions over professional standards, potentially diminishing the role and value of the Code of Ethics in guiding decision making. This shift could have serious consequences, including a weakening of professional accountability, and may obscure pharmacists' responsibility to justify their actions within an established ethical and regulatory framework.

### 4.1 Implications for practice

It is critical to minimize barriers to access and to ensure that patients are adequately informed about the services available to them. Pharmacists who conscientiously object to dispensing certain medications or services should clearly communicate their positions. This transparency will facilitate informed decision-making regarding pharmacy selection for filling prescriptions. In instances of conscientious refusal, patients might be referred to a telehealth service. In the New Zealand context, this occurred with access to early medical abortion on the 1st of November 2022 through regulatory changes. Other jurisdictions would need to consider their patient population needs and funding mechanisms if implementing this.^29^

Isaac et al. (2018) proposes the establishment of a registration system for pharmacies willing to provide specific medications.^25^ Such a publicly accessible register would enhance patient understanding of pharmacy policies, thereby improving access to necessary treatments and reducing ambiguity surrounding provider capabilities. In the New Zealand healthcare context, a variation of this occurred on the 1st November 2022 whereby pharmacies that hold no CO to the provision of early medical abortion services could opt into a prescribing list for medical practitioners to send prescriptions to be filled.^29^ Further research into the effects of such an intervention requires further review.

From a regulatory perspective, defining what CO is and then describing an approach and ongoing management could provide pharmacists with further clarity about where these limits are and expectations about how this is to be managed. As the dialogue and debate surrounding, CRISPR technology, abortifacients euthanasia, physician-assisted dying and future drug technology intensifies, there will be an increasing need for guidance and support for pharmacists.^30^, 31., 32. Global movement in the pharmacist workforce has led to significant cultural diversity within the profession, which may increase the potential for conscientious objections (CO) based on religious and cultural beliefs. In many countries where there is less regulation, these pharmacists could find themselves inadequately supported by existing Codes of Ethics, necessitating a re-evaluation of resources and frameworks to address their unique challenges.^18^ The registration pathway for pharmacists worldwide creates contextual issues if there is less universal recognition about what CO is and how pharmacists should be navigating this.

Given the shortage of pharmacists in some jurisdictions, the implications of sufficient manpower in relation to service provision requires further assessment- the disclosure of CO for a smaller team with less staffing flexibility could have consequences for ongoing service provision if timely access to medications or service is a primary consideration to successful outcome. From a bioethical perspective, a disclosure of CO in a smaller team would affect the principle of beneficence and justice as the inability to provide a service due to CO would create an inequity in terms of access to care or service; more so if there is no reasonable alternative in place, particularly in a rural setting. ^6,12,16, 25,^ The lack of service due to CO creates maleficence if there is no other reasonable or timely manner to refer the request onwards to different care provider, limiting access to health provision. This undermines respect for the patient and contravenes principles of justice, as it may result in unequal access to care based on personal beliefs rather than clinical need.

### 4.2 Implications for future research

The findings from this study indicate a future research agenda. Exploring the real-world implications of CO across the many diverse practice settings will help understand the nuances across the entire pharmacy sector. This will be founded on the need to further examine the cultural context of CO, specifically the role of religion and cultural norms in shaping pharmacists' decisions to exercise CO, particularly in regions where religion influences healthcare delivery.

Document analysis does not allow deep and rich exploration of regional or cultural differences within larger countries (e.g., Canada) and interviews with key stakeholders in these sub-regions should be undertaken to better understand the unique regulations and/or historical contexts influencing CO.

An in-depth analysis of how pharmacists reconcile personal beliefs with professional duties, considering the ethical and legal implications of CO clauses, would be interesting to explore. An examination of the conflict between legal requirements and personal beliefs of pharmacists is missing from the literature and warrants further research.

An exploration of patient autonomy and the ethical dilemmas faced by pharmacists in balancing personal beliefs with professional responsibilities could occur through interview across pharmacy subsectors.

### 4.3 Limitations

The sampling process of OECD countries, FIP member countries and the exclusion criteria to remove non-English papers and legislation means that the data cannot be generalized to the international pharmacy profession. Countries where there was a difference in the regulation of clinical pharmacists and industry pharmacists were also excluded.^18^^,^^19^ The limited focus on these countries means there was less opportunity to capture the experiences and challenges of CO faced by low- and middle-income countries. This under representation of information from these jurisdictions, particularly if there is no regulatory authority to oversee pharmacists creating further challenges in applying the findings of this research considering their unique economic, social and political contexts. ^18^^,^^19^ Documents which reviewed CO separately or utilised legislation but did not appear in a Codes of Ethics were excluded. Codes of Ethics not in English were also excluded.

There are many countries where CO is not addressed or regulated to which our results do not apply. Pharmacy organisations could address CO versus a regulatory body. Document analysis compared what regulatory bodies provided to pharmacists to interpret; no analysis was undertaken to determine what pharmacist's real-world interpretation of CO clauses would be as this was outside the scope of the research aims. Ambiguity in CO clauses, coupled with the absence of clear definitions in guidance documents, may lead to varied interpretations of these documents by pharmacists.

Patient perceptions of healthcare providers that held CO and further exploration of pharmacist real world experiences when navigating CO could be considered as a future direction to add depth to this discussion. Comparing the approaches taken by different jurisdictions in handling patient autonomy—specifically identifying those that require referrals versus those that do not—could reveal how jurisdictions balance personal beliefs with clinical judgement and highlight the potential implications for patient care.

The exploration of cultural influences on the acceptance of CO clauses in a jurisdiction was not undertaken as document analysis is not an appropriate method for this. Cultural contexts significantly shape the interpretation of CO clauses. Societal norms are often influenced by these values and this in turn can impact the scope of services and products within a given jurisdiction. In such jurisdictions where this is implemented, this could be reflective of a dominant social norm for that jurisdiction. Further investigation of how different codes of ethics prioritize patient autonomy and the right to access medication would also strengthen this research.

Despite these limitations, the authors believe that the findings offer valuable insights that warrant further investigation to ensure clarity for pharmacists as they navigate modern day healthcare provision.

## 5 Conclusion

This study aimed to explore international pharmacist Codes of Ethics in relation to CO clauses. As far as the authors are aware this is the first global analysis of this topic, and the findings suggest there are similarities in the Code of Ethics which address CO. However, there continues to be significant differences in some of this guidance and or lack of clear definition of what CO is. There is a requirement for clear, consistent guidance in Codes of Ethics across jurisdictions on the reasonable limits to conscientious objections and how these are mitigated by pharmacists to ensure patients' rights to access treatment are not delayed or impeded. The language around CO and the way in which it is implemented for pharmacists need to be fit for purpose for modern healthcare environments. Understanding how pharmacists use their Code of Ethics when exercising CO in the practice environment could provide insight to professional regulators when updating Code of Ethics for the profession.

## CRediT authorship contribution statement

Conceptualization, SR, L.S.W., J.B., S.L.S, E.B, D.E., J.H, M.H, A.N.,; methodology, S.R.; software, validation, S.R., L.S.W., and XY.L; formal analysis, L.S.W., E.B and S.R.; investigation L.S.W., E.B and S.R.; resources, S.R.; data curation, L.S.W., E.B and S.R.; writing—original draft preparation L.S.W., J.B., E.B and S.R.; writing—review and editing, L.S.W., J.H., XY.L, S.L.S, E.B, D.E., J.B, M.H, A.N; and S.R.; visualization, L.S.W., E.B., S.R.; supervision, S.R. and S.L.S.; project administration,L.S.W.; funding acquisition, PI: SR, S.L.S Research team: L.S.W., S.L.S, JH, D.E., EB, JB, XYL, M.H,A.N.

## Ethics approval

Ethics approval is not required for this review.

## Funding statement

We would like to thank the Faculty Research Development Fund (FRDF) for funding this research project [Grant Number 3727498]. The fund was not involved with the operation of the study nor access to the data. This study was independently run by the School of Pharmacy, University of Auckland.

## Declaration of competing interest

The author(s) declare that there are no conflicts of interest.

## Data Availability

The data presented in this study are available on request from the corresponding author. The data are not publicly available due to ethical and privacy restrictions.
